# Supplementary material for: COVID-19-Associated Pulmonary Aspergillosis, Fungemia, and Pneumocystosis in the Intensive Care Unit: a Retrospective Multicenter Observational Cohort during the First French Pandemic Wave
Source: Microbiol Spectr. 2021 Oct 20;9(2):e01138-21. doi: 10.1128/Spectrum.01138-21 (PMC8528108; doi:10.1128/Spectrum.01138-21)
Supplement: SUPPLEMENTAL FILE 1 — Supplemental material. Download SPECTRUM01138-21_Supp_1_seq8.pdf, PDF file, 0.1 MB [file spectrum01138-21_supp_1_seq8.pdf]

Supplemental Table 1: Description of the 13 patients with two fungal co-infections.

		Infection 1		Infection 2				
Patient #	sex, age	Type (species)	Day after ICU admission	Type (species)	Number of days after first infection	Underlying disease	Antifungal treatment	Outcome (day after diagnosis of first fungal infection)
1	M 73	CAPA (*A. fumigatus*)	D 2	Mucormycosis (*Rhizopus microsporus*)	0	none	L-AmB	Alive (D 21)
2	M 73	CAPA (*A. fumigatus*)	D 13	Fungemia (*Clavispora lusitaniae*)	5	none	Isavuconazole	Alive (D 42)
3	M 40	CAPA (*A. fumigatus*)	D 2	Fongemia *Candida krusei*)	8	none	Voriconazole	Alive (D 43)
4	M 62	CAPA (*A. fumigatus*)	D 6	CA-Fungemia (*Candida albicans*)	7	none	Caspofungin	Alive (D 7)
5	M 76	Mold (*Arthrographis kalrae*)	D 33	Mucormycosis (*Rhizopus arrhizus*)	0	COPD	L-AmB	Deceased (D 49)
6	F 73	CAPA (*A. fumigatus*)	D 12	CA-Fungemia (*Candida albicans*)	6	none	Voriconazole	Deceased (D 6)
7	M 60	CA-Fungemia (*Candida albicans*)	D 13	CAPA (*A. fumigatus*)	14	none	Caspofungin	Deceased (D 19)
8	F 52	CA-Fungemia (*Candida albicans*)	D 10	CA-PCP (*Pneumocystis jirovecii*)	2	none	Micafungin + cotrimoxazole	Deceased (D 6)
9	M 55	CAPA (*A. fumigatus*)	D 11	Mucormycosis (*Rhizopus microsporus*)	0	Auto-HSCT for Lymphoma	L-AmB	Deceased (D 17)
10	M 77	CA-Fungemia (*Candida glabrata*)	D4	CAPA (*A. fumigatus*)	1	none	Voriconazole + caspofungin	Deceased (D 18)
11	M 71	CA-Fungemia (*Candida albicans*)	D 15	CAPA (*A. fumigatus*)	1	none	L-AmB	Deceased (D 6)
12	M 68	CA-Fungemia (*Candida glabrata*)	D8	CAPA (*A. fumigatus*)	1	none	L-AmB + caspofungin	Deceased (D 5)
13	M 61	CAPA (*A. fumigatus*)	D 9	CA-PCP (*Pneumocystis jirovecii*)	0	Renal transplant recipient	L-AmB + cotrimoxazole	Deceased (D 11)

COPD: Chronic obstructive pulmonary disease
HSCT: hematopoietic stem cell transplantation

Supplemental Table 2: Yeast species, delay in occurrence, and associated mortality of the 81 CA-fungemias of the study.

Yeast species	frequency	Delay between ICU hospitalization and positive blood culture median day [IQR 25-75]	Mortality rate n (%)
Candida albicans	48 (59.3%)	14 [12-24.5]	25/48 (52.1%)
Candida parapsilosis	13 (16.0%)	18 [11-30]	3/13 (23.1%)
Candida glabrata	10 (12.3%)	15 [9-19]	7/10 (63.6%)
Clavispora lusitaniae	4 (4.94%)	(15, 20, 28, 31)	0
Candida tropicalis	3 (3.7%)	(12, 17, 44)	2/3 (66.7%)
Candida krusei	1 (1.23%)	10	0
Pichia fabiani	1 (1.23%)	11	0
Saccharomyces cerevisiae	1 (1.23%)	19	0
